# Early Life Adversity and Exposure to Secondhand Smoke among Children: Modeling Risk Health Behaviors and Increasing Environmental Tobacco Exposure

**DOI:** 10.1007/s40653-026-00902-6

**Published:** 2026-05-15

**Authors:** Héctor E. Alcalá, Amanda E. Ng, Nicholas Tkach, Aditya Narayan

**Affiliations:** 1 Department of Behavioral and Community Health, University of Maryland, College Park, College Park, MD 20742 USA; 2 Department of Epidemiology and Biostatistics, University of Maryland, College Park, MD 20742 USA; 3 Department of Psychiatry Stony Brook, Stony Brook University Hospital, New York, NY 11794 USA; 4 Stanford School of Medicine, Stanford, CA 94305 USA; 5 Marlene and Stewart Greenebaum Comprehensive Cancer Center, Program in Oncology, University of Maryland, Baltimore, MD 21201 USA

**Keywords:** Secondhand smoke, Adverse childhood experience, Gender and sexual minorities, Tobacco

## Abstract

This study examined the association between adverse childhood experiences (ACEs), including discrimination due to sexual orientation and gender identity, and exposure to secondhand smoke (SHS). Using data from the 2018–2020 National Survey of Children’s Health (NSCH), logistic regression models calculated adjusted odds ratios (AORs) of two SHS measures: (1) anyone living in the household uses combustible tobacco products, irrespective of them using that product in the home or not, and (2) anyone smoking inside the home. Individual and cumulative ACEs served as independent variables. Data from the 2020 NSCH included discrimination due to sexual orientation and gender identity as an ACE, for children 6–17, but prior years did not. Each additional ACE was associated with a 36% increase in odds of having a smoker in the household (AOR: 1.36; 95% CI: 1.29–1.44) and a 37% increase in odds of smoking occurring in the home (AOR: 1.37; 95% CI: 1.25–1.52). Most ACEs were associated with both SHS outcomes. Experiencing discrimination due to sexual orientation or gender identity was associated with 144% higher odds of smoking occurring in the home (AOR: 2.44; 95% CI: 1.15–5.17). The 2020 NSCH is among the first to include discrimination against sexual and gender minority identity as an ACE. This study highlights that this ACE impacts exposure to tobacco, potentially leading to early modeling of smoking behavior and exposure to the health harming impacts of SHS. Future work should continue to examine discrimination against sexual and gender minority youth as an ACE.

## Introduction

Adverse childhood experiences (ACEs) are potentially harmful exposures which children may experience during childhood (ages 0–17) and typically encompass abuse, neglect and household instability (Felitti et al., 1998). More broadly, ACEs can be conceptualized as stressful, and potentially traumatic, exposures that occur in childhood. These exposures can occur in the home, the neighborhood, or society, making ACEs a multi-level set of experiences. Researchers have argued for taking an ecological approach to help understand exposure to ACEs, where the broad sociopolitical context can help shape the frequency and severity of ACEs (Barajas-Gonzalez et al., 2021). Thus, ACEs can be driven by wider forces like economic conditions, structural racism, structural homophobia and violence.

Research has elucidated the enormous public health and financial burden facing the United States as a result of ACEs. Broadly, ACEs are correlated with lasting, embodied negative effects on the health and well-being of individuals and communities including, but not limited to, increased risks of injury, infection, somatic and behavioral health issues, and incarceration (Dube et al., 2003; Graf et al., 2021; Hughes et al., 2017; Zhang et al., 2022). Beyond the moral and ethical obligation of public health to address these challenges, the economic and social costs to individuals and communities are enormous, with the Centers for Disease Control and Prevention (CDC) identifying that a 10% reduction in ACE exposure in North America would lead to over $56 billion in savings annually (CDC, 2022).

ACEs are associated with increased risk of health harming behaviors, including smoking. For example, adults who experience any ACE are more likely to be current or ever smokers (Alcalá et al., 2016; Vander Weg, 2011). ACEs are hypothesized to be linked with smoking via two mechanisms. First, smoking may comprise the larger environment in which ACEs occur, such that parents or caretakers smoke around a child, modeling this as acceptable behavior (Alcalá et al., 2016). Relatedly, smoking may be associated with ACEs because caretakers in neglecting households may be less likely to curb health harming behaviors, like smoking, among children in their care (Alcalá et al. 2018a, b; Repetti et al. 2002). Second, smoking may be a means of self-medicating (Anda et al., 1999; Boppre & Boyer, 2021; Chandler et al., 2018) to deal with the elevated levels of stress and affective disorders seen among those with a history of ACEs (Anda et al., 1999; Manyema et al., 2018; Nurius et al., 2015).

Smoking may also serve as a considerable behavioral link that mediates the relationship between ACEs and chronic diseases, like cancer (Kelly-Irving et al., 2013). Smoking dramatically increases mortality as a result of smoking-related and smoking-exacerbated illnesses such as heart disease, lung disease, and diabetes (Carter et al., 2015). Smoking also has the potential to impact the health of individuals and communities via secondhand smoke (SHS). Among children and adults, exposure to SHS is associated with elevated risk of respiratory illnesses, lung cancer, cardiovascular disease, sudden infant death syndrome, and ear infections (Health & Services, 2006; Öberg et al., 2011). Globally, rates of SHS exposure among adolescents exceeds 60% (Ma et al., 2021), indicating that SHS remains a pressing health risk. Policy makers have attempted to decrease use of tobacco products via several mechanisms including enacting smokefree policies in multi-unit housing (Pizacani et al., 2012), the workplace (Rashiden, Ahmad Tajuddin, Yee, Zhen, & bin Amir Nordin, 2020) and in schools (Azagba et al., 2016), achieving changes in smoking behaviors and SHS exposure with these policies.

Despite the robust association between ACEs and smoking, minimal scholarship has explored the impact of ACEs on exposure to SHS. Namely only two studies have shown that increased number of ACEs experienced are associated with increased exposure to SHS in the household (Jackson & Testa, 2021; Merianos et al., 2025). However, these studies examined ACEs as interchangeable experiences. That is, ACEs are examined in context of the number of ACEs experienced, or broadly by category of ACE experienced (i.e., household, community or financial) but not with respect to the specific ACE experienced. This is significant given that across a wide variety of outcomes, not all ACEs are associated with all outcomes, or are not associated with all outcomes in the same way (Alcalá et al. 2018a, b; Bloom et al. 2018). In some cases, ACE items have a positive association with an outcome, while other ACEs have a negative association with the same outcome, and variable effect sizes (Alcalá, Mitchell et al., 2018). Thus, we expand on this work by examining the individual and cumulative association of ACEs and environmental/SHS. Further, we extend this work by adding a wider conceptualization of ACEs that includes discrimination due to sexual orientation and gender identity as an ACE, which was not included in some prior work. This addition is important for two reasons: (1) sexual and gender minorities are more likely to report ACEs than their cisgender or heterosexual counterparts, in research that does not include measures of discrimination for their sexual or gender minority status (Andersen & Blosnich, 2013; McCabe et al., 2020; Schnarrs et al., 2019) and (2) sexual and gender minorities use tobacco products at higher rates than their cisgender and heterosexual counterparts (Hoffman et al., 2018; Wheldon et al., 2019). Thus, sexual and gender minority youth may be a particularly vulnerable group in the ACEs and tobacco context, meriting further attention.

## Methods

Data from this study came from the 2018–2020 National Survey of Children’s Health (NSCH).(Child and Adolescent Health Measurement Initiative (CAHMI) (2018), 2020; Child and Adolescent Health Measurement Initiative (CAHMI) (2019), 2021; Child and Adolescent Health Measurement Initiative (CAHMI) (2020), 2022) Since 2016, the NSCH has surveyed households annually about the health of children living in the United States. This web and mail-based survey has parents or guardians of children, answer questions about the health and wellbeing of a child in their household, as well as questions about themselves, and the household more broadly. The NSCH was administered in both English and Spanish, with additional support available through a hotline.

Nine ACEs were included in the 2018–2020 NSCH, and one additional ACE related to discrimination due to sexual orientation or gender identity, was included in 2020 only. As such, analyses were conducted using two different samples: (1) A pooled sample of all responses in 2018–2020, with data on nine ACE items, excluding discrimination due to sexual orientation and gender identity; (2) Responses for children aged 6–17 in 2020, who received all 10 ACE items, including an item on discrimination due to sexual orientation and gender identity. A total of 102,740 surveys were completed as part of the 2018–2020 NSCH (30,530 in 2018, 29,433 in 2019 and 42,777 in 2020). This strategy allowed for us to leverage the large sample size of pooling three years of data, while also creating a subsample that analyzed the new ACE item introduced in 2020. Respondents missing data on any study variable were excluded, resulting in an analytic sample of 94,305 for the 2018–2020 NSCH. Analyses of missing data in the study sample showed that missing cases were more likely to be exposed to SHS and experience ACEs. After limiting the sample to responses of children who were 6–17 and excluding missing data, the analytic sample for the 2020 analyses was 27,823.

## Measures

### Outcome Measurement

The outcomes of interest, exposure to SHS, were operationalized through two binary items asking whether: (1) anyone living in the household uses cigarettes, cigars, or pipe tobacco, irrespective of them using that product in the home or not, and (2) anyone smoking inside the home.

### Exposure Measurement

Adverse Childhood Experiences (ACEs), the exposures of interest, were reported via a series of binary Items asking whether the child had ever: (1) experienced financial hardship, (2) lived with parents/guardians that had divorced or separated, (3) lived with parent/guardian who died, (4) lived with parent/guardian who was jailed, (5) saw or heard adults abuse each other, (6) witnessed or experienced violence in the neighborhood, (7) lived with anyone who was mentally ill, (8) lived with anyone who had problems with drugs or alcohol, (9) experienced racial or ethnic discrimination, or (10) experienced discrimination due to sexual orientation or gender identity. Gender or sexual orientation was only assessed when the selected child was age 6 or older, all other ACE items were assessed of all children.

ACEs were treated in two different ways. First, ACEs were analyzed as individual dichotomous measures, capturing if the ACE was reported or not. Second, ACEs were summed to create a continuous number of ACEs reported. For the pooled 2018–2020 sample this ranged from 0 to 9 ACEs, for the 2020 subsample, that included information on discrimination due to sexual orientation or gender identity, this ranged from 0 to 10 ACEs.

### Potential Confounders

A series of confounders, identified from previous research that also used the NSCH to study ACEs (Alcalá et al. 2022a, b), were included in multivariable analyses. Child level variables included age (as a continuous measure), sex (male or female), race/ethnicity (Latino, White, Black, Asian, Other/multi-racial), insurance status (insured or not insured), having a usual source of care (yes or no), health status (‘excellent, very good or good’, or ‘fair or poor’), and having a health condition in their lifetime (yes or no). Family level variables included primary language spoken in the household (English or other language), household income as a percent of the Federal Poverty Level (FPL; 0–199% FPL, 200–299% FPL, 300–399% FPL, 400%+ FPL), caregiver education level (‘less than college degree’ or ‘college degree or greater’), and family structure (two parents who are currently married, two parents who are not currently married, single parent, or other family structure). The FPL is set by the federal government in United States and is used to determine eligibility for government benefits (Cauthen & Fass, 2008). Survey year (2019, 2020, 2021) was also included as a potential confounder for full sample analyses.

### Analyses

All analyses were conducted using Stata 16, with survey weights to account for complex survey design. Descriptive statistics were calculated for all study variables as appropriate (e.g. means, standard errors, frequencies). Logistic regression models estimated adjusted odds ratios (AORs) and 95% confidence intervals (95% CIs) and p-values, adjusted for confounders. In analyses, ACEs were considered individually (e.g. each specific ACE in its own model) and cumulatively (i.e. total number of ACEs) as exposure/independent variables. Separate models were also constructed for each of the SHS outcomes. Analyses were done for the pooled 2018–2020 data that included children of all ages and then repeated for 2020, while being limited to children ages 6–17.

## Results

Weighted descriptive statistics are presented in Table 1, for both the 2018–2020 sample and the 2020 subsample. Characteristics were similar between the 2018–2020 and 2020 samples. Approximately 14% of children lived in a household with a smoker, but smoking in the household was relatively rare (1.8% in 2018–2020, 2.1% in 2020). Children had one ACE on average (mean = 1.1). The majority of the sample was male (2018–2020: 51.2%; 2020: 51.3%), White (2018–2020: 51.8%; 51.5%), U.S.-born (2018–2020: 95.9%; 2020: 94.5%), insured (2018–2020: 94%; 2020: 93.4%), had a usual source of care (2018–2020: 76.7%; 2020: 75.9%), had excellent, very good, or good health (2018–2020: 98.6%; 2020: 98.3%), and lacked a health condition (2018–2020: 62.0%; 2020: 54.3%). The majority or plurality of children also primarily spoke English in the home (2018–2020: 86.1%; 2020: 85.1%), lived within 0–199% of the FPL (2018–2020: 38.9%; 2020: 38.6%), had caregivers with a college degree or greater (2018–2020: 51.5%; 2020: 50.1%), and had two currently married parents (2018–2020: 65.1%; 2020: 64.7%).

**Table 1:** Sample characteristics, national survey of children’s health

Variable	2018–2020 Sample Ages 0–17 (*N* = 94,305)		2020 Sample Ages 6–17 (*N* = 27,823)	
	% or Mean	SE	% or Mean	SE
*Someone in the household is a smoker*	14.15%	0.25%	14.38%	0.46%
*Smoking happens in the household*	1.79%	0.08%	2.06%	0.17%
*Adverse childhood experiences*				
Experienced financial hardship	46.17%	0.36%	44.70%	0.67%
Lived with parents or guardians divorced or separated	22.76%	0.30%	28.27%	0.62%
Lived with parent or guardian who died	2.81%	0.11%	3.32%	0.21%
Lived with parent or guardian who was jailed	6.90%	0.18%	7.94%	0.37%
Saw or heard adults abuse each other	5.39%	0.18%	6.03%	0.35%
Witnessed or experienced violence in neighborhood	3.96%	0.15%	5.31%	0.37%
Lived with anyone who was mentally ill	8.18%	0.19%	9.57%	0.38%
Lived with anyone who had problems with drugs or alcohol	8.29%	0.20%	10.35%	0.43%
Experienced racial or ethnic discrimination	4.61%	0.16%	6.81%	0.35%
Experienced discrimination due to sexual orientation or gender identity	--	--	1.40%	0.14%
Number of adverse childhood experiences (continuous)	1.09	0.01	1.24	0.02%
Number of adverse childhood experiences (categorical)				
0	41.55	0.35	38.33	0.64
1	33.03	0.34	31.06	0.63
2	13.26	0.25	15.66	0.53
3	5.43	0.16	6.54	0.32
4 or more	6.72	0.19	8.41	0.38
*Child’s sex*				
Boy	51.21%	0.36%	51.34%	0.67%
Girl	48.79%	0.36%	48.66%	0.67%
*Child’s age*	8.64	0.04	11.55	0.05
*Child’s race/ethnicity*				
Latino	24.83%	0.39%	25.28%	0.76%
White	51.80%	0.35%	51.48%	0.68%
Black	12.42%	0.25%	12.75%	0.46%
Asian	4.57%	0.13%	4.63%	0.25%
Other race or multiracial	6.39%	0.15%	5.86%	0.27%
*Child’s nativity status*				
US born	95.88%	0.15%	94.49%	0.33%
Foreign born	4.12%	0.15%	5.51%	0.33%
*Child’s health insurance status*				
Not insured	5.96%	0.21%	6.64%	0.41%
Insured	94.04%	0.21%	93.36%	0.41%
*Child has usual source of care*				
No	23.35%	0.34%	24.12%	0.64%
Yes	76.65%	0.34%	75.88%	0.64%
*Child’s health status*				
Excellent, very good or good	98.56%	0.10%	98.34%	0.18%
Fair or poor	1.44%	0.10%	1.66%	0.18%
*Child has or had a health condition*				
No	61.97%	0.34%	54.33%	0.66%
Yes	38.03%	0.34%	45.67%	0.66%
*Primary language spoken at home*				
Other	13.94%	0.33%	14.63%	0.68%
English	86.06%	0.33%	85.37%	0.68%
*Household poverty level*				
0–199% of Federal Poverty Level	38.90%	0.37%	38.63%	0.70%
200–299% of Federal Poverty Level	16.51%	0.26%	17.60%	0.53%
300–399% of Federal Poverty Level	12.44%	0.21%	12.38%	0.39%
400% or more of Federal Poverty Level	32.15%	0.30%	31.39%	0.55%
*Highest education level of child’s caregivers*				
Less than college degree	48.51%	0.36%	49.86%	0.67%
College degree or greater	51.49%	0.36%	50.14%	0.67%
*Family structure*				
Two parents who are currently married	65.13%	0.35%	64.73%	0.67%
Two parents who are not currently married	8.29%	0.23%	6.55%	0.41%
Single parent	21.58%	0.30%	24.13%	0.59%
Other family structure	5.00%	0.18%	4.59%	0.28%
*Survey Years*				
2018	33.41%	0.34%	--	--
2019	33.43%	0.35%	--	--
2020	33.16%	0.32%	--	--

Table 2 presents adjusted odds ratios of SHS exposure among children aged 0–17 in the full 2018–2020 sample. In fully adjusted models, all ACEs were related to higher odds of having a smoker in the household. The strongest association was observed for those who lived with anyone who had problems with drugs or alcohol (AOR: 2.72; 95% CI: 2.39–3.09). When operationalized as a continuous number, each additional ACE was related to a 36% increase in odds of having a smoker in the household (AOR: 1.36; 95% CI: 1.31–1.40). Similarly, all ACEs except living with a parent or guardian who died, were associated with higher odds of smoking occurring in the home. The strongest association was observed for those living with parent or guardian who was jailed (AOR: 2.44; 95% CI: 1.87–3.18). Each additional ACE was associated with a 30% increase in odds of smoking occurring in the home (AOR: 1.30; 95% CI: 1.23–1.38).

**Table 2:** Odds of smoking exposure, children ages 0–17, by adverse childhood experience, national survey of children’s health, 2018–2020 (*N* = 94,305)

Variable	Someone in Household is a Smoker			Smoking Occurs in Home		
	AOR	95% CI	*P*-Value	AOR	95% CI	*P*-Value
Experienced financial hardship	1.80	(1.65, 1.97)	< 0.0001	1.88	(1.49, 2.38)	< 0.0001
Lived with parents or guardians divorced or separated	1.42	(1.27, 1.59)	< 0.0001	1.39	(1.08, 1.80)	0.012
Lived with parent or guardian who died	1.28	(1.05, 1.57)	0.017	1.36	(0.99, 1.89)	0.07
Lived with parent or guardian who was jailed	2.55	(2.22, 2.94)	< 0.0001	2.44	(1.87, 3.18)	< 0.0001
Saw or heard adults abuse each other	2.17	(1.82, 2.59)	< 0.0001	1.95	(1.46, 2.62)	< 0.0001
Witnessed or experienced violence in neighborhood	1.79	(1.49, 2.15)	< 0.0001	1.87	(1.37, 2.56)	< 0.0001
Lived with anyone who was mentally ill	1.99	(1.74, 2.27)	< 0.0001	1.87	(1.44, 2.43)	< 0.0001
Lived with anyone who had problems with drugs or alcohol	2.72	(2.39, 3.09)	< 0.0001	2.25	(1.77, 2.86)	< 0.0001
Experienced racial or ethnic discrimination	1.53	(1.25, 1.87)	< 0.0001	1.85	(1.15, 2.96)	0.01
Number of adverse family experiences	1.36	(1.31, 1.40)	< 0.0001	1.30	(1.23, 1.38)	< 0.0001

Each AOR represents a separate model. Models adjusts for child level variables (age, sex, race/ethnicity, insurance status, having a usual source of care, health status, and having condition in their lifetimes), family level variables (primary language spoken in the household, income as a percent of the federal poverty level, care giver educational level, and family structure), and survey year

AOR= Adjusted odds ratio

Table 3 presents adjusted odds ratios of SHS exposure among the subsample of children aged 6–17 in 2020. After full adjustment, all ACEs except living with a parent or guardian who died, or experiencing discrimination due to sexual orientation or gender identity, were related to higher odds of having a smoker in the household. The strongest association was observed for those witnessing or experiencing violence in the neighborhood, (AOR: 2.46; 95% CI: 1.73–3.49). For the continuous sum of ACEs, each additional ACE was associated with a 36% increase in odds of having a smoker in the household (AOR: 1.36; 95% CI: 1.29–1.44). All ACEs were associated with higher odds of smoking occurring in the home except for living with a parent or guardian who died and experiencing racial or ethnic discrimination. Importantly, experiencing discrimination due to sexual orientation or gender identity was associated with more than double the odds of smoking occurring in the home (AOR: 2.44; 95% CI: 1.15–5.17). Each additional ACE was related to a 37% increase in odds of smoking occurring in the home (AOR: 1.37; 95% CI: 1.25–1.52).

**Table 3:** Odds of smoking exposure, children ages 6–17, by adverse childhood experience, national survey of children’s health, 2020 (*N* = 27,823)

Variable	Someone in Household is a Smoker			Smoking Occurs in Home		
	AOR	95% CI	*P*-Value	AOR	95% CI	*P*-Value
Experienced financial hardship	1.78	(1.49, 2.10)	< 0.0001	2.16	(1.45, 3.21)	< 0.0001
Lived with parents or guardians divorced or separated	1.67	(1.38, 2.03)	< 0.0001	1.59	(1.05, 2.41)	0.028
Lived with parent or guardian who died	0.93	(0.69, 1.24)	0.616	1.58	(0.94, 2.64)	0.083
Lived with parent or guardian who was jailed	2.6	(2.01, 3.36)	< 0.0001	2.54	(1.56, 4.12)	< 0.0001
Saw or heard adults abuse each other	2.5	(1.89, 3.30)	< 0.0001	2.77	(1.61, 4.77)	< 0.0001
Witnessed or experienced violence in neighborhood	2.46	(1.73, 3.49)	< 0.0001	2.53	(1.39, 4.59)	0.002
Lived with anyone who was mentally ill	1.78	(1.40, 2.27)	< 0.0001	1.91	(1.28, 2.86)	0.002
Lived with anyone who had problems with drugs or alcohol	2.38	(1.91, 2.98)	< 0.0001	2.72	(1.86, 3.98)	< 0.0001
Experienced racial or ethnic discrimination	1.61	(1.17, 2.23)	0.004	1.45	(0.89, 2.36)	0.138
Experienced discrimination due to sexual orientation or gender identity	1.63	(0.96, 2.78)	0.068	2.44	(1.15, 5.17)	0.021
Number of adverse family experiences	1.36	(1.29, 1.44)	< 0.0001	1.37	(1.25, 1.52)	< 0.0001

Each AOR represents a separate model. Models adjusts for child level variables (age, sex, race/ethnicity, insurance status, having a usual source of care, health status, and having condition in their lifetimes) and family level variables (primary language spoken in the household, income as a percent of the federal poverty level, care giver educational level, and family structure)

## Discussion

This study is one of the few that has examined the association between ACEs and SHS exposure. Consistent with prior studies (Jackson & Testa, 2021; Merianos et al., 2025), we find that ACEs are associated with higher SHS exposure. However, we expand on prior work through disaggregation of individual ACE exposures, and comparing results that include or exclude an ACE item on discrimination due to sexual orientation and gender identity. In the full sample, all ACEs increased odds of exposure to SHS. However, in the limited sample, not all ACEs were associated with both SHS outcomes. This highlights the importance of analyzing ACE items individually. Failing to do so not only obscures individual associations, but also provides less information which may be leveraged to guide future investigation or intervention. For example, the interventions to address the impacts of parental separation might be different from interventions to address neighborhood violence. Further, designing a study to further examine the impact of discrimination would look different from a study that examines poverty. Finally, we demonstrate that the cumulative burden of ACEs is associated with SHS, highlighting the importance of examining both individual ACEs and cumulative exposures. This was the case in analyses that included or excluded the ACE item on discrimination due to sexual orientation and gender identity.

Notably, in the 2020 data, living with a parent or guardian who died was not found to be significantly associated with either outcome. We hypothesize that parental loss can be correlated with the source of SHS itself in some cases, thus diminishing potential associations between ACEs. Specifically, if a parent is a smoker, and the source of SHS, losing that parent prematurely, could make this association weaker. Additionally, because parental loss was a rare ACE exposure, it is possible that the subsample lacks the power to detect this association.

This study is among the first to examine discrimination due to sexual orientation and gender identity, as an ACE in relation to SHS exposure. Importantly, we show that this type of discrimination was associated with increased SHS exposure, suggesting increased environmental risks and sources of modeling of smoking behavior for queer youth. This increased exposure to modeling of smoking behavior may be a contributing factor to use of tobacco products among lesbian, gay, bisexual and transgender (LGBT) youth, who use tobacco at higher rates than their non-LGBT counterparts (Gentzke et al., 2022; Johnson et al., 2019). Further, LGBT youth may engage in tobacco use behaviors as a means of coping with discrimination, and its impacts (Kelly et al., 2015; Ong et al., 2021). Importantly, discrimination due to sexual orientation and gender identity are interpersonal level manifestations of larger systems of oppression (i.e., heterosexism and cissexism), highlighting how ACEs can capture multi-level stressors that have impacts on health.

Importantly, there are many different experiences that can be captured by the measure of discrimination due to sexual orientation and gender identity captured in the NSCH, which impact how findings from this study should be interpreted. Specifically, children, may choose not to disclose their experiences with LGBT discrimination because their parents may not yet be aware of their sexual orientation or gender identity. As a result, rates of discrimination due to sexual orientation or gender identity, may be underestimated by parents or guardians because they are unaware that they are happening. Additionally, it is possible that some of the reported discrimination against sexual orientation or gender identity is targeting cisgender, heterosexual children, who are either perceived as being a sexual or gender minority by parents or others. Thus, the impact of anti-LGBT discrimination can extend more broadly to include those not identifying as LGBT.

Overall, our study highlights that ACEs are correlated with exposures to other environmental hazards. In the case of SHS, this expands the potential ways in which ACEs are related to tobacco. Instead of only viewing tobacco use as a behavioral coping mechanism to deal with ACEs, future work should consider that the connection between ACEs and tobacco can begin earlier in a child’s life. Specifically, as potential behavior that is modeled by adults in the household, and as part of exposure to harmful SHS. In fact, given that substance use is already an ACE item, it might be that exposure to SHS should be examined as an ACE item. We echo calls by other researchers to expand the conceptualization of ACEs (Afifi, 2020; Choi et al., 2020; Cronholm et al., 2015; Karatekin & Hill, 2019). More broadly, the association of ACEs and SHS suggests a need to examine other potential harmful factors in the home environment that occur alongside ACEs, including exposure to other environmental hazards like pests in the household, lack of clean water and exposure to air pollution. Finally, it is important to consider how the broader policy environment can meaningfully impact the health of vulnerable children, like those with a history of ACEs. In particular, smokefree policies in indoor setting can limit exposure to SHS and improve health (Frazer et al., 2016), can help lessen the deleterious impacts of ACEs.

Although this study has numerous strengths, it is not without limitations. One limit of the present study is classification bias secondary to underreporting. Parents may underreport due to fear of being perceived negatively, or because they may not be with a child to witness an ACE happening. This may be particularly salient when considering ACEs that can happen outside of the home, like discrimination, which may mean parents/guardians may be unaware of their child’s experiences. In fact, even in retrospective self-reporting of ACEs, bias towards underreporting has been documented (Hardt & Rutter, 2004). Further, when parent and child reporting of ACEs has been compared, children report ACEs at higher rates, suggesting that the NSCH suffers from systematic underreporting (Ng et al., 2025). Next, analyses could not account for important covariates, particularly a child’s own sexual orientation or gender identity, which could impact study findings. Additionally, because missingness was associated with both ACEs and SHS, we suspect that our findings will underestimate the strength of the associations because respondents with ACEs or SHS exposure are more likely to have been dropped in analyses. Lastly, as the study is cross-sectional, temporality cannot be established.

Despite limitations, our study helps to expand how tobacco use and exposure can operate in the context of ACEs. Namely, there may be multiple paths that link ACEs to tobacco that include use of tobacco and exposure to SHS. Disentangling these pathways will require more detailed examinations of tobacco exposure, as well as potentially considering SHS exposure an ACE. Future research can build upon this study by examining associations between ACEs and SHS using longitudinal data, to provide additional context for the timing, severity, and duration of both the exposure and outcome. Further, clinicians who encounter children with a known history of ACEs should consider that these children may be at greater risk for SHS exposure, and potentially benefit from education about the harms of smoking, in an effort to prevent the uptake of smoking modeled in their environment. Given that ACEs are hypothesized to increase smoking via self-medicating to deal with stress, it would also be helpful for children with a known history of ACEs to understand healthy ways to deal with stress. Lastly, our findings support including measures of anti-LGBT discrimination as ACE items. Future work can reexamine the association between ACEs and health outcomes and determine if and how inclusion of anti-LGBT discrimination impacts these findings.

## Data Availability

Data can be downloaded from the National Survey of Children’s Health: https://www.childhealthdata.org/learn-about-the-nsch/NSCH.
